# Effects of Ketamine Infusion on Oxygenation in Patients with Chronic Obstructive Pulmonary Disease Undergoing Lung Cancer Surgery

**DOI:** 10.5152/TJAR.2023.21683

**Published:** 2023-02-01

**Authors:** Feride Karacaer, Ebru Biricik, Murat Ilgınel, Demet Laflı Tunay, Oya Baydar, Alper Avcı, Hakkı Ünlügenç

**Affiliations:** 1 Department of Anaesthesiology and Reanimation, Çukurova University Faculty of Medicine, Adana, Turkey; 2 Department of Pulmonary Disease, Çukurova University Faculty of Medicine, Adana, Turkey; 3 Department of Thoracic Surgery, Çukurova University Faculty of Medicine, Adana, Turkey

**Keywords:** Chronic obstructive pulmonary disease, ketamine, one-lung ventilation, oxygenation, thoracic anaesthesia

## Abstract

**Objective:**

Ketamine changes respiratory mechanics, provides airway relaxation, and alleviates bronchospasm in patients with pulmonary disease. This study investigated the effect of a continuous infusion of ketamine during thoracic surgery on arterial oxygenation (PaO_2_/FiO_2_) and the shunt fraction (*Q*_s_/*Q*_t_) in patients with chronic obstructive pulmonary disease.

**Methods:**

Thirty patients older than 40 years, diagnosed with chronic obstructive pulmonary disease, and undergoing lobectomy were recruited for this study. Patients were allocated randomly to 1 of 2 groups. At the induction of anaesthesia, group K received intravenous (iv) 1 mg kg^-1^ ketamine as a bolus and followed by 0.5 mg kg^-1^ h^-1^ infusion until the end of the operation. Group S received the same amount of 0.9% saline as a bolus at induction and followed by a 0.5-mL kg^-1^ h^-1^ infusion of 0.9% saline until the end of the operation. PaO_2_ and PaCO_2_ values, FiO_2_ levels, PaO_2_/FiO_2_ ratio, peak airway pressure (*P*_peak_), plateau airway pressure (*P*_plat_), dynamic compliance, and shunt fraction (*Q*_s_/*Q*_t_) were recorded during two-lung ventilation as a baseline and at 30 (one-lung ventilation, OLV-30) and 60 (OLV-60) minutes during one-lung ventilation.

**Results:**

PaO_2_, PaCO_2_, PaO_2_/FiO_2_ values, and *Q*_s_/*Q*_t_ ratio were similar between the 2 groups at OLV-30 minute (*P* = .36, *P* = .29, *P* = .34). However, at OLV-60 minute, PaO_2_, PaO_2_/FiO_2_ values were significantly increased, and *Q*_s_/*Q*_t_ ratios were significantly decreased in group K than in group S (*P* = .016, *P* = .011, *P* = .016).

**Conclusions:**

Our data suggest that a continuous infusion of ketamine and desflurane inhalation in patients with chronic obstructive pulmonary disease during one-lung ventilation increase arterial oxygenation (PaO_2_/FiO_2_) and decrease shunt fraction.

**Main Points**

- Chronic obstructive pulmonary disease is defined by chronic airflow limitation, incompletely reversible.
- One-lung ventilation applied for lung resection produces gas exchange abnormalities and increased intrapulmonary shunt, deepening hypoxemia in chronic obstructive pulmonary disease patients during surgery.
- Ketamine changes respiratory mechanics, provides airway relaxation, and alleviates bronchospasm in patients with pulmonary disease.
- The continuous infusion of ketamine in patients with chronic obstructive pulmonary disease undergoing lung cancer surgery during one-lung ventilation increases arterial oxygenation (PaO_2_/FiO_2_) and decreases shunt fraction.

## Introduction

Chronic obstructive pulmonary disease (COPD) is defined by chronic airflow limitation, incompletely reversible. Pathologic changes include obstructive bronchiolitis in the small airways, parenchymal destruction, reduced elastic recoil due to emphysema, endothelial dysfunction, and arterial stiffening in the pulmonary vasculature.^1^ Additionally, inflammation and narrowing of peripheral airways lead to decreased forced expiratory volume in 1 second (FEV1) and FEV1/FVC (forced vital capacity [FVC]) ratio and results in hypoxemia and hypercapnia due to ventilation-perfusion (VA/*Q*) mismatch.^2^

One-lung ventilation (OLV) during thoracic surgery is a frequently chosen technique to provide ideal surgical exposure. However, during OLV, intrapulmonary shunt and dead space increase because of continued perfusion of the non-dependent lung and insufficient expansion of the dependent lung. Anaesthetics and the lateral decubitus position may also increase the shunt ratio and inhibit hypoxic pulmonary vasoconstriction (HPV).^3^ Furthermore, conventional methods such as high external positive end-expiratory pressure (PEEP) and high peak pressures may deteriorate oxygenation and respiratory mechanics during OLV because of high intrinsic PEEP (PEEPi) and the development of air trapping in patients with COPD. Additionally, the individual response of patients with COPD to external PEEP cannot be predicted.^4^

Ketamine has been reported to change respiratory mechanics, provide airway relaxation, and alleviate bronchospasm in patients with the pulmonary disease by blocking the reuptake of catecholamines into presynaptic sympathetic neurons,^5^ and decreasing the production of inflammatory cytokine in pulmonary tissues.^6^ Continuous infusion of ketamine in patients with asthma has been reported to improve gas exchange and chest compliance significantly.^7^

In the literature, although many agents (dexmedetomidine, inhalation agents, propofol) have been proposed to improve gas exchange and oxygenation during OLV, as far as we know, no studies have evaluated the effect of ketamine during thoracic surgery on arterial oxygenation and the shunt fraction in patients with COPD undergoing lung cancer surgery.^8,9^

This study was undertaken to investigate the effect of a continuous infusion of ketamine during thoracic surgery on arterial oxygenation (PaO_2_/FiO_2_) and the shunt fraction (*Q*_s_/*Q*_t_) in patients with COPD undergoing lung cancer surgery. We hypothesised that ketamine infusion during thoracic surgery would significantly improve oxygenation and decrease the shunt fraction in patients with COPD undergoing lung cancer surgery.

## Method

This prospective, randomised, double-blinded, controlled study was approved by the Ethical Committee of Çukurova University Medical Faculty (IRB = 56/6), and written informed consent was obtained from all subjects participating in the trial. The trial was registered prior to patient enrolment at clinicaltrials.gov (NCT02962999). The study was performed at the Department of Anesthesiology at the Hospital of Çukurova University Medical Faculty, Adana, Turkey. Forty-two patients undergoing lobectomy due to lung cancer were recruited in this study. The inclusion criteria included age over 40 years, American Society of Anesthesiologists (ASA) physical status II to III, and patients diagnosed with COPD according to the Global Strategy for the Diagnosis, Management, and Prevention of Chronic Obstructive Lung Disease (GOLD) criteria.^1^ Patients with heart and/or respiratory failure (PaO_2_ <55 mmHg, PaCO_2_ >55 mmHg), severe liver or kidney disease, or obesity [body mass index (BMI) ≥30 kg m^-2^] were excluded from the study. Demographic variables (age, height, and weight) and BMI values were evaluated and recorded preoperatively. The categories of COPD, medications used, comorbidities, preoperative spirometry data, duration of OLV and operation, right/left lobectomy ratios, and the number of cigarettes used by patients in the groups were also recorded.

### Monitoring

Standard monitoring in the operating room included lead II-V electrocardiogram (ECG), pulse oximetry (SpO_2_), non-invasive blood pressure (NIBP), end-tidal CO_2_ pressure (ETCO_2_), bispectral index (BIS), core temperature, urine output, and ventilator settings such as tidal volume, respiratory rate, peak airway pressure (*P*_peak_), plateau airway pressure (*P*_plat_), and dynamic compliance. Ventilator settings were adjusted based on pulse oximetry and measurements of arterial-blood gas analyses.

### General Anaesthesia

Two intravenous accesses with an 18-gauge peripheral intravenous catheter were established in all patients. General anaesthesia was induced with propofol (Propofol Ampul, Fresenius Kabi, Copenhagen, Denmark), fentanyl (Fentanyl Ampul, Johnson & Johnson, NJ, ABD), and rocuronium (Esmeron Ampul, Merck Sharp & Dohme, NJ, ABD) and maintained using 4%-6% desflurane (Suprane, Baxter, Ill, ABD) + 60% air + 40% O_2_ mixture and a continuous infusion of remifentanil (Ultiva flakon, GlaxoSmithKline, Brentford, England). Patients were monitored using the BIS index to titrate the desflurane concentration and the level of anaesthesia; we aimed to keep a BIS index between 40 and 60. If the BIS index increased above 60, the concentration of desflurane was increased. Patients were intubated with a double-lumen tube (Covidien, Dublin, OH, ABD), and the position of the tube was confirmed using a fiberoptic bronchoscope (Olympus, Hamburg, Germany). After induction of anaesthesia, the radial artery was catheterised to monitor continuous arterial pressure and analyse blood gas samples, and a central venous catheter was placed in the right internal jugular vein to evaluate central venous pressure.

### Randomisation

Fifteen minutes before induction of anaesthesia, the patients were allocated randomly to 1 of 2 groups. A computer-generated random number table was used for randomisation. An anaesthetist, blinded to the study groups, prepared 2 syringes and 2 solutions containing ketamine (5 mg mL^-1^, Ketalar flakon, Pfizer, New York, ABD) or 0.9% saline. Both syringes and solutions were labelled “study drug” and coded to maintain the double-blind nature of the study. Ketamine was supplied in 10 mL vials at a 50-mg mL^-1^ concentration and diluted with 100 mL normal saline to a 5 mg mL^-1^ concentration. The placebo saline solution was prepared similarly.

At induction of anaesthesia, group K (n = 15) intravenously (iv) received 1 mg kg^-1^ ketamine (2 mL in total) as a bolus, followed by 0.5 mg kg^-1^ h^-1^ infusion until the end of the operation. Group S (n = 15) received the same amount of iv bolus but with 0.9% saline (2 mL in total) at induction, followed by 0.5 mL kg^-1^ h^-1^ infusion of 0.9% saline until the end of the operation.

### Mechanical Ventilation

Following endotracheal intubation with double-lumen tubes, patients were ventilated with volume-controlled ventilation (VCV), tidal volume (TV) 8 mL kg^-1^, ratio of inspirium:expirium (I:E) = 1:2.5, and PEEP = 5 cmH_2_O in both groups. Respiratory frequency was set to maintain an end-tidal carbon dioxide partial pressure in 5-6 kPa. *P*_peak_ was limited to 30 cmH_2_O. During OLV, the dependent lung was ventilated with VCV, TV 5 mL kg^-1^, I:E = 1:2.5, PEEP = 5 cmH_2_O. Fraction of inspired oxygen (FiO_2_) was 40% initially and then adjusted to maintain SaO_2_ at 92% to 98%. In the event of desaturation (SaO_2_ <92%), FiO_2_ was increased step by step from 0.4 to 1.0 in 0.2 increments. If desaturation persisted despite the therapy above, the ventilation strategy was converted to TLV. Respiratory frequency during OLV was also set to maintain an end-tidal carbon dioxide partial pressure in 5-6 kPa.

### Follow-Up

Haemodynamic [heart rate (HR) and mean arterial pressure (MAP)] and respiratory variables (SpO_2_, EtCO_2_) were continuously monitored but recorded only just before the initiation of OLV (baseline) and at 30 (OLV-30) and 60 (OLV-60) minutes during OLV. PaO_2_ and PaCO_2_ values, FiO_2_, PaO_2_/FiO_2_ ratio, *P*_peak_, *P*_plat_, static compliance, and the *Q*_s_/*Q*_t_ ratio were recorded just before the initiation of OLV (as baseline) and at OLV-30 and OLV-60 minutes during OLV. Dynamic compliance was calculated using the following formula: 

Dynamic compliance = tidal volume/(P_peak_ - PEEP)

The shunt fraction was calculated with a formula suggested by Koessler et al^10^ as follows:

*Q*_s_/*Q*_t_ = (5.8 × RI) + 6.7 

RI = respiratory index 

RI = (PAO_2_ - PaO_2_)/PaO_2_

PAO_2_ = alveolar O_2_ pressure

PAO_2_ = ([PB - PH_2_O] × FiO_2_) - PaCO_2_

PB = atmosphere pressure = 760 mmHg

PH_2_O = water vapour pressure = 47 mmHg

All patients were followed-up routinely in the postoperative care unit (PACU) postoperatively. Patients were monitored regarding haemodynamic and respiratory variables to evaluate the postoperative pulmonary complications. PaO_2_, PaCO_2_, and PaO_2_/FiO_2_ values were measured routinely only at 20 minutes after arrival to the PACU to evaluate pulmonary function status. It was planned that if the patients experienced signs of dyspnoea or episodes of hypoxia (SpO_2_ <90%), they would be transferred to the intensive care unit (ICU). Patients were evaluated for 72 h postoperatively for the occurrence of pneumonia and atelectasis on X-ray and the beginning of acute lung injury (ALI). All other complications such as hypotension and atrial fibrillation were recorded after arrival in the ICU. Length of hospital stay and mortality rates at 30 days were recorded.

### Definitions

In addition to clinically detected new or progressive lung infiltration on X-ray, 2 or more symptoms were regarded as pneumonia; fever >38°C or hypothermia, leucocytosis or leucopenia, and purulent secretion.^11^ A PaO_2_/FiO_2_ ratio <300 mmHg, diffuse pulmonary infiltration at 72 h, and exclusion of hydrostatic pulmonary oedema because of other primary causes were defined as ALI.^12^

### Study Outcomes

The primary outcome was accepted as improvement in oxygenation expressed as the PaO_2_ and the PaO_2_/FiO_2_ ratio. The secondary outcome was regarded as shunt fraction, postoperative pneumonia, atelectasis, ALI, and the length of hospital stay.

### Power Analysis

Power analysis for testing the hypothesis and determining sample size used a primary endpoint defined as improvement in oxygenation. Considering Rees and Gaines's^13^ study in which the effect of ketamine on the PaO_2_ was investigated, the effect size was defined as 1.25, and it required 15 subjects per study group for a power of 0.9 and a significance level of .05. This study calculated the effect size assuming that PaO_2_ values would be 176 ± 31 mmHg in group K and 142 ± 22 mmHg in group S at 30 minutes. Therefore, the sample size was determined by supposing that the PaO_2_ values at 30 minutes would be 34 mmHg higher in group K.

### Statistical Analysis

All analyses were performed using the IBM Statistical Package for Social Sciences Version 20.0 (IBM Corp.; Armonk, NY, USA) statistical software package. Categorical variables are expressed as numbers and percentages, and continuous variables are summarised as mean and standard deviation or median and minimum-maximum where appropriate. The Fisher exact test was used to compare categorical variables between the groups. To compare continuous variables between 2 groups, Student’s *t*-test or the Mann–Whitney *U*-test was used depending on whether the statistical hypotheses were fulfilled. Repeated measures analysis of variance was applied to evaluate the change in the measurements obtained in the time interval. The normality of distribution for continuous variables was confirmed with the Shapiro–Wilk test. The statistical level of significance for all tests was *P* <.05. 

## Results

**Figure 1.**
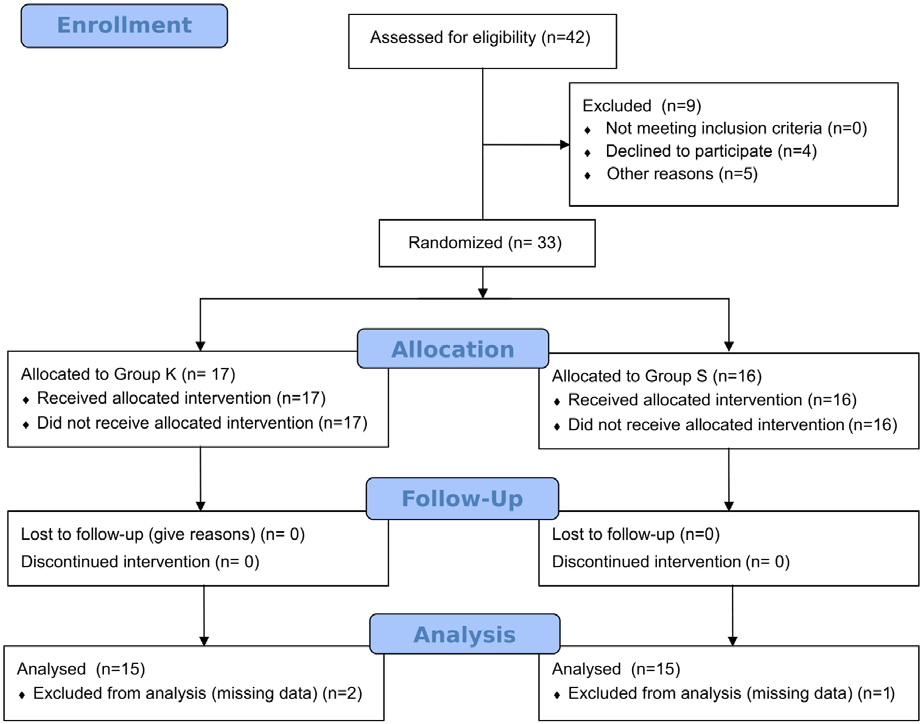
Consort of flow diagram.

Forty-two patients undergoing lobectomy due to lung cancer were recruited in this study and randomised into the ketamine and saline groups. However, 12 patients were excluded from the study; 4 patients declined to participate, 3 because of missing data, and 5 due to changes in the surgical plan (Figure 1). Therefore, 30 patients (15 for each group) completed the study. There was no significant difference between the 2 groups regarding demographic variables and BMI values (Table 1). The categories of COPD, preoperative spirometry data, duration of OLV and operation, right/left lobectomy ratios, and the number of cigarettes used by the patients in the groups were also similar (Table 1).

Intraoperative respiratory and haemodynamic variables are presented in Table 2. There were no significant differences between the 2 groups concerning haemodynamic variables (HR, MAP). FiO_2_, PaCO_2_, *P*_peak_, *P*_plat_, and dynamic compliance values at baseline and OLV-30 and OLV-60 minutes were similar between the 2 groups (Table 2).

**Figure 2.**
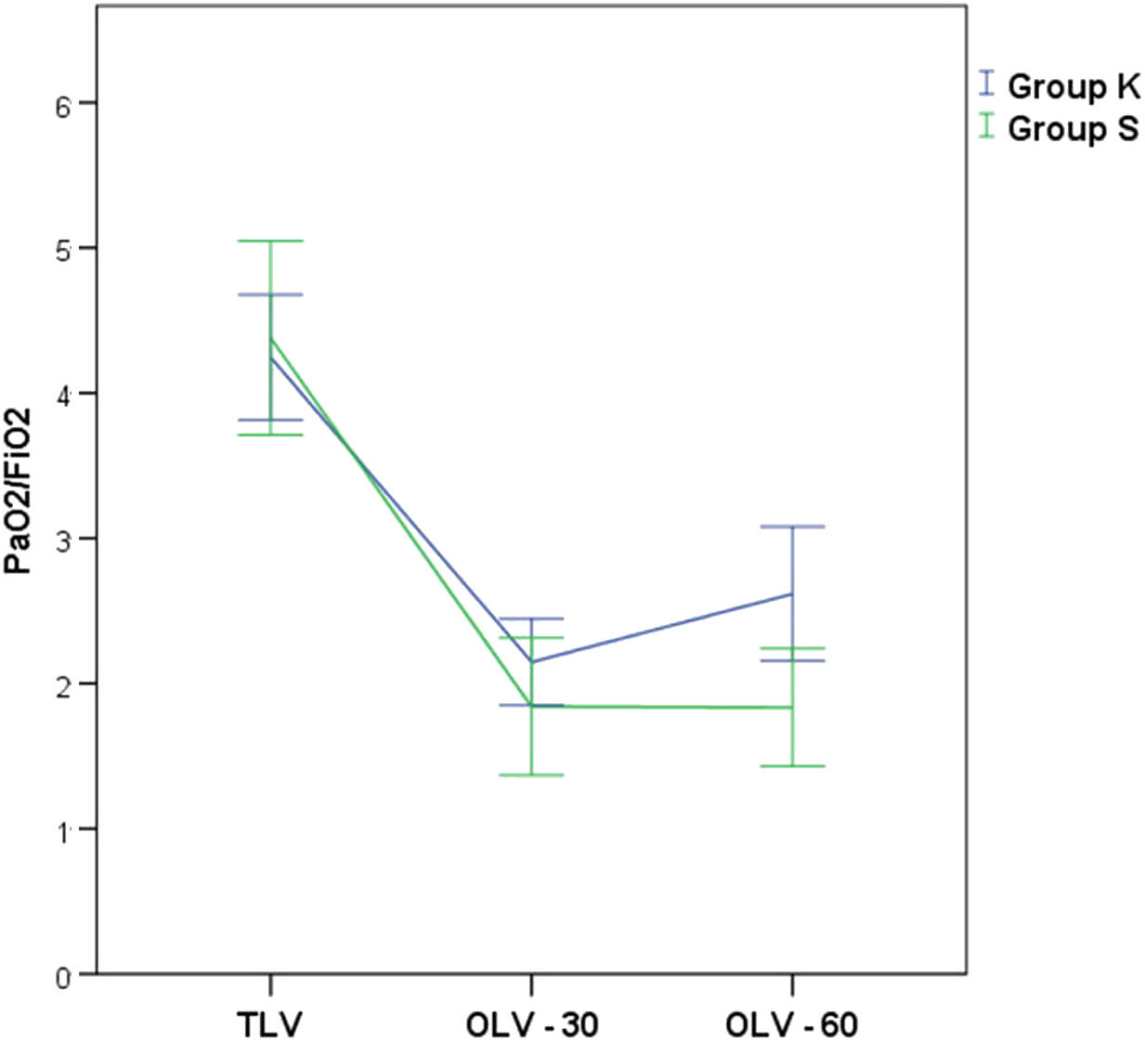
PaO_2_/FiO_2_ ratios. Group K, ketamine group; group S, saline group; TLV, two-lung ventilation; OLV-30, 30 minutes after start of the one-lung ventilation; OLV-60, 60 minutes after start of the one-lung ventilation.

**Figure 3.**
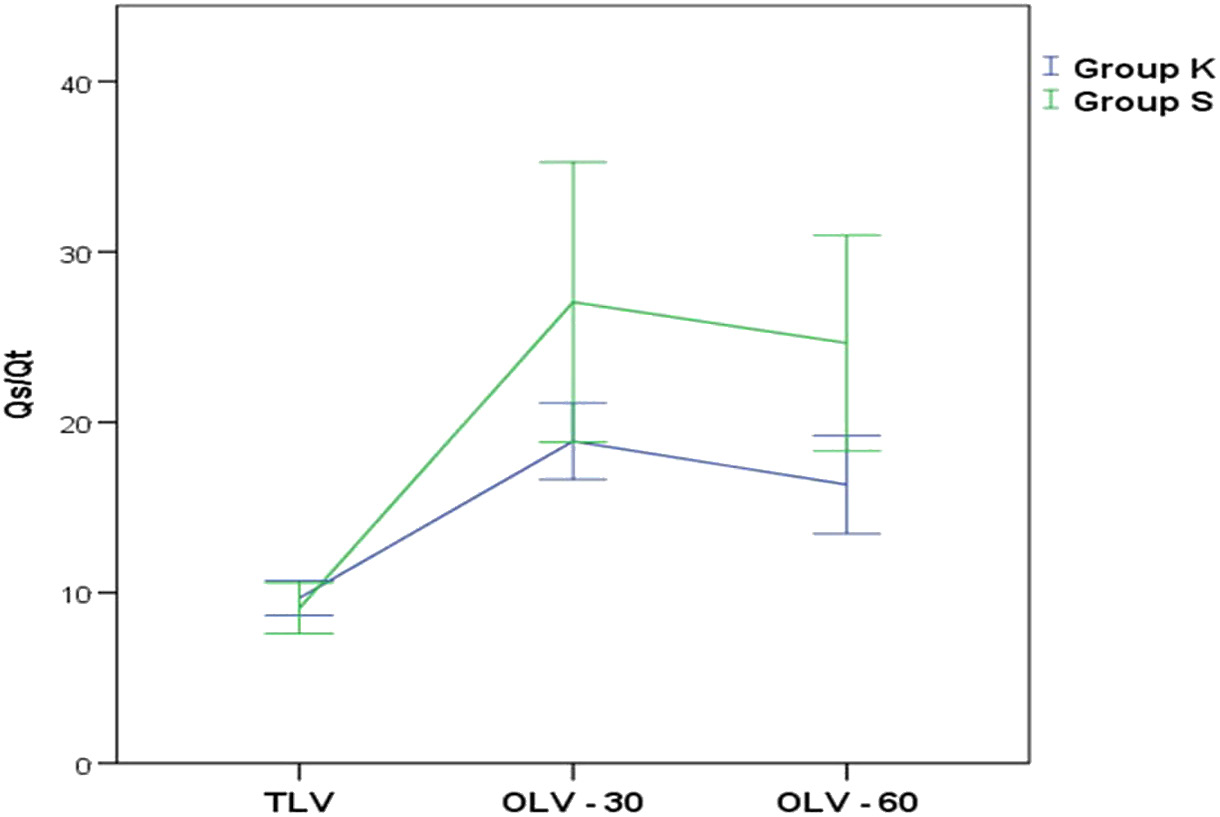
Shunt fraction ratios. Group K, ketamine group; group S, saline group; TLV, two-lung ventilation; OLV-30, 30 minutes after start of the one-lung ventilation; OLV-60, 60 minutes after start of the one-lung ventilation.

PaO_2_, PaO_2_/FiO_2_, and the *Q*_s_/*Q*_t_ ratio were similar between the 2 groups at OLV-30 minutes (*P* = .36, *P* =.29, *P* = .34). However, at OLV-60 minutes, PaO_2_ and PaO_2_/FiO_2_ values were significantly increased, and *Q*_s_/*Q*_t_ ratios were significantly decreased in group K compared with group S (*P* = .016, *P* = .011, *P* = .016, Table 2 and Figures 2 and 3).

The over time changes of the measurements were examined and PaO_2_ and PaO_2_/FiO_2_ values significantly increased from the 30th to the 60th minute in group K (*P *= .031 for PaO_2_, *P* = .049 for PaO_2_/FiO_2_). However, there was not a statistically significant variation in group S (*P* = .999 for both).

Postoperative data are presented in Table 3. In the PACU, PaO_2_ and the PaO_2_/FiO_2_ ratio at the postoperative 20th minute were similar, and there was no significant difference between the 2 groups.

One patient was transferred to the ICU in group K because of tachypnoea, dyspnoea, and O_2_ desaturation (SaO_2_ <90%) after extubation. In group S, 2 patients were transferred to the ICU without extubation because of insufficient respiratory effort after anaesthesia was turned off, and 1 patient was re-intubated in the PACU because of dyspnoea and O_2_ desaturation.

Although statistically not significant, 6 complications (atelectasis in 3 patients and ALI in 3 patients) were detected in group S during the follow-up period in the ICU, whereas none in group K. The length of hospital stay was found similar between the 2 groups.

## Discussion

This study demonstrated that continuous infusion of ketamine and desflurane inhalation increases arterial oxygenation (PaO_2_/FiO_2_) and decreases shunt fraction in patients with COPD during OLV. In the present study, higher PaO_2_ and PaO_2_/FiO_2_ values were comparable to previously reported PaO_2_ levels in patients using intraoperative ketamine during OLV.^14^ However, Rees and Gaines^13^ reported significantly higher PaO_2_ levels with ketamine than we found.

Chronic obstructive pulmonary disease is characterised by expiratory airflow limitation because of small airway inflammation (obstructive bronchiolitis), air trapping, and parenchymal destruction. These factors adversely affect the *V*/*Q* matching and lung mechanics, increase the bronchial smooth muscle tone, and change pulmonary vasculature.^15^ Luminal narrowing of pulmonary arterioles and air-trapping hyperinflation in patients with COPD increase the right-to-left shunt ratio (*Q*_s_/*Q*_t_ ratio) and causes hypoxemia and hypercapnia. Furthermore, OLV applied for lung resection produces gas exchange abnormalities and increased intrapulmonary shunt, deepening hypoxemia and oxygen desaturation (<90%) during surgery.^16,17^ This level of desaturation raises concern that cellular organ function may be impaired or damaged due to reduced oxygen delivery.^18^ The interventions for improving oxygenation during OLV include an increase in the inspired fraction of oxygen followed by the recruitment of both lungs, PEEP application to the ventilated lung, and continuous positive airway pressure application to the nonventilated lung.^18,19^ However, complex physiology, diversity in patients’ underlying conditions, and the limitation of PEEP application to overcome the *V*/*Q* mismatch can render these attempts ineffective.^20^ Various therapeutic agents such as β-agonists or dexmedetomidine have been available in such situations, but limited studies support their use.^8^ Therefore, treatment options for hypoxemia in these patients during OLV are limited. Ketamine has been reported to produce bronchodilation by blocking both *N*-methyl d-aspartate receptor-induced bronchoconstriction and reuptake of catecholamines into presynaptic sympathetic neurons. Furthermore, it acts as a relaxant agent on bronchial smooth muscles by vagal inhibition and improves pulmonary compliance.^5^ In the present study, ketamine infusion decreased *Q*_s_/*Q*_t_ ratios and improved arterial oxygenation (PaO_2_/FiO_2_) at OLV-60 minutes in patients with COPD during OLV.

Hypoxic pulmonary vasoconstriction is less protective in patients with COPD due to a decrease in endothelium-dependent relaxation, leading to decreased pulmonary artery compliance and increased pulmonary artery stiffness.^21^ In the present study, ketamine was chosen to increase PaO_2_/FiO_2_ values and decrease the *Q*_s_/*Q*_t_ ratio. Previous studies demonstrated that ketamine acted as a relaxant agent on bronchial smooth muscles by vagal inhibition, improved pulmonary artery compliance, decreased *V*/*Q* mismatch, and provided haemodynamic stability.^13,14,22,23^ However, conflicting results have been presented in the literature. Ogawa et al^24^ investigated the effects of etomidate and ketamine on pulmonary vasorelaxation in response to receptor-dependent and independent endothelial activators and found that both etomidate and ketamine selectively attenuated receptor-mediated endothelium-dependent pulmonary vasorelaxation by inhibiting the nitric oxide and endothelium-derived hyperpolarising factor components of the response. Conflictingly, Weinreich et al^14^ reported that intrapulmonary shunting and arterial oxygen desaturation during OLV were decreased with ketamine anaesthesia, and cardiovascular stability improved during OLV. That study differed from ours because they had no patients with COPD, and the effect of ketamine was primarily attributed to its positive inotropic and chronotropic effect. However, contrary to Weinreich et al’s^14^ trial, the increased oxygenation and decreased shunt fraction in our study were attributed to the bronchodilatory effect of ketamine because there was no significant difference in haemodynamic variables between the two groups.

The efficiency of ketamine on pulmonary vasculature and perfusion is still under investigation. In some recent clinical studies, ketamine exhibited an ideal effect in patients with pulmonary hypertension with no increase in pulmonary vascular resistance (PVR).^25^ The present study could not demonstrate any beneficial effect of ketamine on PVR because we could not measure pulmonary arterial pressure and PVR.

During the period of OLV in the lateral position, numerous factors influence arterial oxygenation. In addition to the increased right-to-left shunt through the non-dependent lung, the alveolar pressure in the dependent lungs may also affect arterial oxygenation.^21,22,24-26^ Previous studies of COPD mainly focused on hyperinflation and PEEPi because of air trapping during OLV.^4,26,27^ However, there is controversy regarding PEEPi and oxygenation during OLV in patients with COPD. Some authors support a negative correlation between PEEPi and oxygenation,^4^ whereas others advocate that PEEPi increases functional residual capacity and improves oxygenation in severe COPD.^28^ However, this statement should be considered according to the severity of COPD. In this study, we were not able to measure PEEPi but administered a longer expiration period (I/E: 1/2.5) than usual, which would decrease the possibility of PEEPi. In addition, we rarely observed decreased compliance and increased *P*_peak_ and *P*_plat_ levels because most of our patients had mild-to-moderate COPD. Nevertheless, the effects of ketamine on respiratory mechanics cannot be underestimated, and the results of this study cannot be extrapolated.

Ketamine has long been used in treating patients with acute asthma as a bronchodilator and in managing anaesthesia.^5,6,9^ Huber et al^7^ demonstrated that ketamine was usually effective in reducing airway resistance in patients with existing pulmonary dysfunction, involving slightly, moderately, or severely increased airway resistance. In the present study, we observed no increased airway resistance in any patients with COPD, and this might be related to the relief of bronchospasm of ketamine evidenced by lower *P*_plat_ and *P*_peak_ levels, although nonsignificant. However, Howton et al^29^ evaluated ketamine as a bronchodilator in patients with severe acute asthma and found no difference between the ketamine and placebo groups. Accordingly, ketamine was concluded to have had no bronchodilatory effect, despite satisfying or beneficial effects.

The present study has several limitations. First, although *P*_plat_ and *P*_peak_ values were better, we failed to demonstrate any significant difference between the 2 groups, mainly due to the relatively small sample size. Second, we were not able to measure PEEPi. Lastly, the dose of ketamine might vary. In adults, the induction dose of ketamine has been reported as 1-2 mg kg^-1^ iv, and it should be given at a rate of around 0.1-0.5 mg kg^-1^ h^-1^ to maintain anaesthesia without producing significant cumulative effects.^30^ However, when used for refractory bronchospasm in operating rooms and refractory status asthmaticus in ICUs, a loading dose of 0.1-0.2 mg kg^-1^ is used, followed by an infusion of 0.15-2.5 mg kg^-1^ h^-1^.^5^ We aimed to evaluate the effects of ketamine on oxygenation and respiratory mechanics during OLV; we used a 1-mg kg^-1^ bolus dose of ketamine, followed by 0.5 mg kg^-1^ h^-1^ continuous infusion; however, lower or higher doses of ketamine could also be used.

## Conclusion

Continuous infusion of ketamine (0.5 mg kg^-1^ h^-1^) and desflurane inhalation in patients with COPD undergoing lung cancer surgery during OLV increases arterial oxygenation (PaO_2_/FiO_2_) and decreases shunt fraction. Although *P*_plat_ and *P*_peak_ values were better in the ketamine group, we failed to demonstrate any significant effect on these respiratory mechanics. Further studies with a more significant patient number are needed to demonstrate this effect.

## Figures and Tables

**Table 1.** Patients’ Demographic Data and Operative Data

	**Group K (n = 15)**	**Group S (n = 15)**	***P***
Age (year)	66.47 ± 8.51	63.13 ± 7.35	**.261 ^a^**
Height (cm)	172.4 ± 9.9	167.8 ± 6	**.136 ^a^**
Weight (kg)	71.9 ± 15.5	76.3 ± 9.8	**.361 ^a^**
BMI (kg m^-2^)	23.9 ± 3.27	24.8 ± 2.66	**.341 ^a^**
COPD categories			
Gold A	10 (66.7%)	13 (86.7%)	**.477 ^b^**
Gold B	4 (26.7%)	1 (6.7%)	
Gold C	1 (6.7%)	1 (6.7%)	
Smoking, mean ± SD	43.33 ± 14.48	38.33 ± 15.77	**.367 ^c^**
Median (25-75 PCT)	50 (30-50)	35 (30-50)	
Preoperative spirometry			
FEV1 (lt)	2.12 ± 0.4	1.93 ± 0.5	**.130 ^a^**
FEV1 (%)	73.4 ± 16.45	65.2 ± 3.2	**.136 ^a^**
FVC (lt)	3.54 ± 0.69	3.03 ± 0.69	**.055 ^a^**
FVC (%)	93.2 ± 15.1	81.7 ± 18.5	**.071 ^a^**
FEV1/FVC (%)	60 ± 6.9	62.6 ± 5.6	**.274 ^a^**
Residual volume	116.4 ± 36.9	117.9 ± 64.2	**.939 ^a^**
Diffusion	57.4 ± 12.9	61 ± 17.8	**.530 ^a^**
Diffusion/VA	60.1 ± 12.2	63.5 ± 12.4	**.456 ^a^**
TLC	89.3 ± 27.9	85.3 ± 26.9	**.697 ^a^**
Operation time (min)	180.7 ± 38.9	183.3 ± 41.5	**.857 ^a^**
OLV time (min)	114.4 ± 32.3	118.1 ± 34.4	**.842 ^a^**
Right/left lobectomy	**10/5**	**11/4**	**.999 ^b^**
Right upper lobe	**6 (40%)**	6 (40%)	
Right middle lobe	**1 (7%)**	**1 (7%)**	
Right lower lobe	**3 (20%)**	**4 (27%)**	
Left upper lobe	**2 (13%)**	**2 (13%)**	
Left lower lobe	**3 (20%)**	**2 (13%)**	

BMI, body mass index; COPD, chronic obstructive pulmonary disease; FEV1, forced expiratory volume in 1 second; FVC, forced vital capacity; OLV, one lung ventilation; TLC, total lung capacity; VA, alveolar volume. *P < .05.

^a^ *t*-test.

^b^Fisher exact test.

^c^Mann–Whitney *U*-test.

**Table 2.** Intraoperative Data

	Group K (n = 15)	Group S (n = 15)	*P*
HR (beats min^-1^)			
TLV	89.4 ± 17.6	79.3 ± 13.1	**.082 ^a^**
30 min OLV	82.2 ± 14.9	78.5 ± 9.7	**.424 ^a^**
60 min OLV	80 ± 14.6	73.1 ± 9.6	**.138 ^a^**
MAP (mmHg)			
TLV	94.1 ± 18.9	91.3 ± 11.2	**.628 ^a^**
30 min OLV	81.5 ± 11.9	83.2 ± 16.9	**.749 ^a^**
60 min OLV	81.1 ± 13.5	74.8 ± 11.8	**.184 ^a^**
PaCO_2_ (mmHg)			
TLV	40.2 ± 4.7	38.9 ± 5.1	**.467 ^a^**
30 min OLV	41.1 ± 11.5	43 ± 7.4	**.676 ^a^**
60 min OLV	41.1 ± 7.4	42.7 ± 5.7	**.517 ^a^**
PaO_2_ (mmHg)			
TLV	195 (149-226)	175 (123-210)	**.561 ^b^**
30 min OLV	104 (77.9-138)	96 (72.9-134)	**.359 ^b^**
60 min OLV	128 (97.7-190)	79.1 (73.3-100)	.016*^,^^b^
PaO_2_/FiO_2_			
TLV	430 (373-480)	473 (334-520)	**.272 ^b^**
30 min OLV	197 (168-255)	181 (113-268)	**.290 ^b^**
60 min OLV	273 (194-343)	161 (123-250)	.011*^,^^b^
*P*_peak_ (cmH_2_O)			
TLV	25.6 ± 5	27.4 ± 5	**.341 ^a^**
30 min OLV	29.9 ± 6.5	32.2 ± 6.4	**.340^a^**
60 min OLV	30.8 ± 6.4	33.3 ± 6.2	**.276^a^**
*P*_plato_ (cmH_2_O)			
TLV	21.8 ± 3.7	22.3 ± 4.4	**.722^a^**
30 min OLV	24.9 ± 6.4	26.5 ± 5.7	**.480^a^**
60 min OLV	25.6 ± 6	27.5 ± 2	**.412^a^**
Compliance (mL cmH_2_O^-1^)			
TLV	38.4 ± 12.2	40.6 ± 9.5	**.592^a^**
30 min OLV	20.6 ± 6.5	22 ± 7.9	**.599^a^**
60 min OLV	19.8 ± 6.8	20.2 ± 6.1	**.865^a^**

HR, heart rate; MAP, mean arterial pressure; OLV, one-lung ventilation; *P*_peak_, peak inspiratory airway pressure; *P*_plat_, end-inspiratory airway pressure; PaCO_2_, partial arterial carbon dioxide pressure; PaO_2_, partial arterial oxygen pressure; PaO_2_/FiO_2_, partial arterial oxygen pressure/fraction of inspired oxygen; TLV, two-lung ventilation. *P <.05.

^a^*t*-test.

^b^Mann-Whitney *U*-test.

**Table 3.** Postoperative Data

	Group K	Group S	*P*
PaO_2_/FiO_2_ at PACU	247.7 ± 128.8	260.5 ± 96.9	**.31^a^**
Need for ICU	1 (7%)	3 (20%)	**.59^b^**
Atelectasis	0 (0%)	3 (20%)	**.22^b^**
Acute lung injury	0 (0%)	3 (20%)	**.22^b^**
Length of hospital stay (day)	7.4 ± 2.5	8.5 ± 4.4	**.39^b^**

ICU, intensive care unit; PACU, postoperative care unit. *P < .05

^a^*t*-test.

^b^Fisher exact test.
